# Inhibition of TMUB1 blocks apoptosis and NF‐κB pathway‐mediated inflammation in recurrent spontaneous abortion

**DOI:** 10.1002/iid3.879

**Published:** 2023-05-26

**Authors:** Xiuping Zhang, Yuanjing Hu, Zhiping Zhang, Xueluo Zhang, Lixia Liang, Xiangrong Cui, Yuanxia Wu, Fen Hu, Xueqing Wu

**Affiliations:** ^1^ Reproductive Medicine Center Children's Hospital of Shanxi and Women Health Center of Shanxi Taiyuan Shanxi China; ^2^ Department of Gynecologic Oncology Tianjin Central Hospital of Gynecology Obstetrics Tianjin China

**Keywords:** apoptosis, inflammation, NF‐κB, recurrent spontaneous abortion, TMUB1

## Abstract

**Introduction:**

Approximately 50% of cases with recurrent spontaneous abortion (RSA) have unexplained etiology. Aberrant expression of transmembrane and ubiquitin‐like domain containing 1 (TMUB1) is closely related to a series of diseases, including RSA. However, the function and underlying mechanism of TMUB1 in the occurrence of RSA has not been described.

**Methods:**

TMUB1 expression was detected in the placental villous tissues of 30 women with normal miscarriages and 12 women with RSA. The pregnant mice were injected intraperitoneally with lipopolysaccharide (LPS) to induce abortion. Human chorionic trophoblast cells were treated with LPS. Pathological analysis of placental tissues was performed by hematoxylin and eosin staining.

**Results:**

TMUB1 was highly expressed in the placental villous tissues of RSA patients compared to the patients who underwent induced abortions. After LPS administration, the mice exhibited high embryo absorption and pathological alterations, as well as presented an increase in inflammation and apoptosis (the etiology of RSA induction) in placental tissues. Moreover, the upregulated expression of TMUB1 was also found in placental tissues of LPS‐induced mice, and further investigation showed that TMUB1 deficiency blocked embryo loss as well as inhibited apoptotic rate and inflammation after LPS activation. Furthermore, we found that the loss of TMUB1 suppressed the phosphorylation of IkappaB kinase (IKK) α/β and attenuated cytoplasmic‐nuclear translocation of nuclear factor‐κB (NF‐κB) p65 in LPS‐induced cells.

**Conclusion:**

Our results indicate that TMUB1 may involve in the modulation of apoptosis and NF‐κB pathway‐mediated inflammation in RSA. Therefore, TMUB1 may develop as a potential biomarker for RSA treatment.

## Graphical abstract

Our results indicate that transmembrane and ubiquitin‐like domain containing 1 (TMUB1) may involve in the modulation of apoptosis and nuclear factor‐κB (NF‐κB) pathway‐mediated inflammation in recurrent spontaneous abortion (RSA).

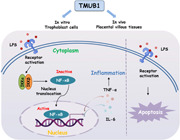

## 1 INTRODUCTION

Recurrent spontaneous abortion (RSA) is defined as the spontaneous loss of two or more consecutive idiopathic miscarriages before 24 weeks,^1^ and is deemed as one of the most common obstetrical complications. The main causes of RSA are uterine structural defects, abnormal embryo development, infectious inflammation, disorders of the immune system, and endocrine problems.^2^, ^3^ Despite miscarriages occurring in 15% of clinically diagnosed pregnancies, the overall rate of reproductive losses is close to 50%.^4^, ^5^ However, 50% of patients with RSA have no obvious cause, and these cases are called unexplained RSA.^6^ Therefore, it is very necessary to study the in‐depth mechanism of RSA occurrence and development.

Inflammation is a manifestation of innate immunity, a fundamental protective response that is conserved in all multicellular animals.^7^ In many cases of RSA, an inflammatory response is usually observed at the maternal‐fetal interface as the final pathological assault.^8^ Inflammatory processes at the feto–ematernal interface can take place due to the invasion of micro‐organisms, and a sterile maternal immune reaction against alloantigens on the fetus or trophoblast.^9^, ^10^ In the early stages of normal pregnancy, pro‐inflammatory T helper cytokine (Th) 1 stimulates angiogenesis, which is essential for embryo implantation.^8^ However, long‐term exposure to Th1 cytokines can induce an immune response at the cellular level, which adversely affects the fetus and leads to miscarriage.^9^ Pregnancy induces sterile inflammation, which is common in injury, the endometrium during menstruation, and tumor occurrence.^8^ In addition, the etiology of RSA is variable, in which unbalanced placental apoptosis plays an important role.^11^ These findings indicate that the occurrence of RSA is closely related to apoptosis and inflammatory response. Therefore, it is urgent to elucidate the mechanism of local immunomodulatory regulation of the maternal‐fetal interface and how to improve recurrent abortion.

Transmembrane and ubiquitin‐like domain‐containing 1 (TMUB1), also known as HOPS, is encoded by a gene identified during a screening of liver regeneration.^12^ TMUB1 is characterized by a ubiquitin‐like domain and three trans‐membrane leucine‐rich regions.^13^ Studies have shown that TMUB1 plays an important role in inflammatory response and apoptosis. For example, TMUB1 can inhibit the degradation of p53 and promote mitochondria‐induced apoptosis.^14^ Moreover, TMUB1 promotes apoptosis by interacting with tumor protein 63 in hepatocellular carcinoma cells.^15^ In macrophages, TMUB1 knockdown reduces lipopolysaccharide (LPS)‐induced inflammatory responses, while TMUB1 overexpression accelerates the degradation of the inhibitor of nuclear factor‐κB (NF‐κB) alpha (IκBα) and the phosphorylation of NF‐κB p65.^16^ However, it is unknown whether TMUB1 plays an inflammatory and apoptotic role in RSA.

Based on RNA sequencing analysis that TMUB1 expression is significantly upregulated in the placental villous tissues of RSA patients compared with normal induced abortion patients,^17^ we guessed that recurrent miscarriages could be affected by TMUB1. In this study, we confirmed that TMUB1 was highly expressed in the villous tissues of RSA women and LPS‐induced mice. Then, the trophoblast cells and mouse models induced by LPS were used to explore the possible role of TMUB1 in abortion.

## 2 MATERIALS AND METHODS

### 2.1 Patients and tissue specimens

Thirty women who underwent elective termination of normal pregnancies were recruited for the clinical study. According to the inclusion and exclusion criteria, 12 women with RSA were selected from the 40 women to detect TMUB1 expression. The “Control” (*N* = 30) and “RSA” (*N* = 12) samples were collected from the placental villous tissues of patients with normal abortion and RSA patients, respectively. All tissue samples were collected between 6 and 12 weeks of gestation in 2021. General clinical information of patients was listed in Table 1. The study was carried out following the ethical principles of the latest version of the Declaration of Helsinki. Each participant provided informed consent in the study.

**Table 1:** Baseline characteristics of patients in the control group and the recurrent spontaneous abortion (RSA) group.

Group	Control	RSA
Number	30	12
Maternal age (year), (mean ± SD)	32.75 ± 3.31	30.2 ± 4.54
Gestational age (week), (mean ± SD)	7.67 ± 0.89	6.07 ± 0.74
Number of previous miscarriage, *n* (mean ± SD)	0	2.42 ± 0.79
≥3 miscarriages, *n* (%)	–	3 (25)
Miscarriage ≥ 10 weeks, *n* (%)	–	1 (8.3)

Abbreviation: SD, standard deviation.

Inclusion criteria were (1) pregnant females aged 18 to 45 years, and a history of RSA. (2) the current pregnancy had to be confirmed by a clinician. (3) normal karyotypes of both partners of the RSA couples. (4) the ability to give informed consent. (5) negative for hepatitis B, hepatitis C, syphilis, AIDS, or other infectious diseases.

Exclusion criteria were (1) pregnant women with chronic hypertension or chronic antithrombotic therapy during pregnancy. (2) the presence of chromosomal abnormalities, and uterine malformation. (3) recent upper respiratory tract infection. (4) systemic illness (eg, eclampsia, asthma, antiphospholipid syndrome, or allergies). (5) endocrine disease.

### 2.2 Animals and groups

BABL/C mice were purchased from Changsheng Biotechnology, and the mice aged 6‐8 weeks were allowed free access to food and water in a controlled environment for 1 week. LPS‐induced abortion models were established as mentioned previously.^18^ Briefly, female mice (*n* = 18) were housed overnight with males (*n* = 9) at a ratio of 2:1. It was considered pregnant if there was milky white or light‐yellow jelly (vaginal plug) on the next morning, and all female mice were pregnant in our experiments. On Day 7 of pregnancy, the pregnant mice were divided into the Control group (*n* = 9) and the LPS group (*n* = 9). In the mice experiments with TMUB1 knockdown, the pregnant mice (*n* = 60) were randomly divided into four groups (Control, LPS, LPS + Lv‐shNC, LPS + Lv‐shTMUB1, *n* = 15 in each group). The statistical experiments on abortion rate were carried out by each group of 9 or 15 mice, and the rest of the experiments were conducted in six mice of each group.

### 2.3 Lentiviral transduction and virus infection

Lentiviral plasmids containing shRNA sequences targeting TMUB1 (Lv‐shTMUB1) and the negative control shRNA fragments (Lv‐shNC) were generated. One day before LPS administration, the pregnant mice were injected with 200 μL of lentivirus (1 × 10^6^ TU/mL) in the tail vein, and the control group was injected with the same volume of saline.

### 2.4 LPS‐induced abortion model

Mice in the LPS group were injected intraperitoneally (I.P.) with 0.15 μg/g LPS to induce abortion, and mice in the control group were given the same volume of normal saline. On Day 9 of pregnancy, the mice were killed peacefully. The uterus was exposed under sterile conditions, then the embryos and placental tissues (including decidua basalis from the bottom decidua to the labyrinth region) of mice were removed to photograph and calculate the embryo resorption rate and absorption rate. The pathological changes of placental villi were observed in mice treated with LPS and the control group. All experimental procedures that involved animals were conducted under the National Research Council's Guide for the Care and Use of Laboratory Animals. Ethical approval was obtained from the animal ethics institute of Shanxi medical university (201922021).

### 2.5 Cell culture and transfection

Human chorionic trophoblast cells (HTR‐8/Svneo) were purchased from Zhong Qiao Xin Zhou Biotechnology (Shanghai, China). The cells were cultured with the Roswell Park Memorial Institute 1640 (RPMI‐1640) medium containing 10% fetal bovine serum (FBS) in an incubator at 37°C and 5% CO_2_. HTR‐8/Svneo cells were transfected with TMUB1‐specific small interfering RNA (siRNA) or the negative control (siNC) using Lipofectamine 3000 (Invitrogen) according to the manufacturer's protocol. After 24 h‐transfection, the cells were treated with LPS (100 ng/mL) for 24 h.

### 2.6 Quantitative real‐time polymerase chain reaction (qRT‐PCR)

The messenger RNA (mRNA) levels of tumor necrosis factor‐α (TNF‐α), interleukin‐6 (IL‐6), and TMUB1 were determined by qRT‐PCR. Total RNAs were prepared using TRIpure (BioTeke, China) and reverse‐transcribed using BeyoRT II M‐MLV reverse transcriptase (Beyotime, China). Quantitative PCR was performed using SYBR Green dye (Solarbio, China) to measure amplification on a fluorescence quantitative PCR instrument (Exicycler 96, Bioneer). The sequences of primers were as follows (5′–3′): IL‐6 forward (mus): ATGGCAATTCTGATTGTATG, and reverse (mus): GACTCTGGCTTTGTCTTTCT; TMUB1 forward (mus): GTAGGCGATGAGGTGACTGT, and reverse (mus): GCTGTGCTGGTGTTGTGG; TNF‐α forward (mus): CAGGCGGTGCCTATGTCTCA, and reverse (mus): GCTCCTCCACTTGGTGGTTT; TMUB1 forward (Homo): CCTCGTGCTACGGCTGAAA, and reverse (Homo): GTTGGGAGGGAGGTGAAGG; IL‐6 forward (homo): GTCCAGTTGCCTTCTCCC, and reverse (Homo): GCCTCTTTGCTGCTTTCA; TNF‐α forward (Homo): GAGTGACAAGCCTGTAGCC, and reverse (Homo): AAGAGGACCTGGGAGTAGAT.

### 2.7 Protein extraction and western blot

Whole‐cell lysates were collected in protein lysis buffer (Beyotime), and the protein concentration was determined by a bicinchoninic acid (BCA) protein assay kit (Beyotime). The protein samples were separated by electrophoresis (sodium dodecyl sulfate‐polyacrylamide gel electrophoresis) and then transferred to polyvinylidene difluoride membranes (Millipore). Subsequently, the membranes were incubated with primary and secondary antibodies in TBST containing 5% milk. Primary antibodies against phosphorylation‐p65 (p‐p65, Rabbit, ABclonal), p65 (Rabbit, ABclonal), IkappaB kinase (IKK) α (IKKα, Rabbit, Affinity), phosphorylation‐IKKα/β (p‐IKKα/β, Rabbit, Affinity), and TMUB1 (Rabbit, Abcam) were included in the study. Histone H3 antibody (Mouse, ABGENT) and β‐actin antibody (Mouse, Santa Cruz Biotechnology) were used to normalize the protein expression. Western blots were developed using ECL detection reagents (Beyotime).

### 2.8 Immunohistochemistry (IHC)

Paraffin‐embedded sections from placental tissues were dewaxed and rehydrated. Then endogenous peroxidases were inhibited by 3% H_2_O_2_ for 30 min at room temperature. Slices were subsequently blocked with 1% BSA and incubated with TMUB1 antibody (Abcam) overnight at 4°C and then incubated with an HRP‐labeled secondary antibody anti‐rabbit IgG (ThermoFisher) for 1 h at 37°C. Finally, the sections were counterstained with DAB (MXB Biotechnologies), and DNA was counterstained with hematoxylin (Solarbio). Tissues were imaged using a microscope (400X magnification).

### 2.9 Haematoxylin and eosin (H&E) staining

Slides were deparaffinized in xylenes (Aladdin), rehydrated sequentially in ethanol (5 min in 95%, 5 min in 85%, and 5 min in 75%), and washed for 2 min in water twice. The sections were stained with hematoxylin for 5 min, followed by staining with eosin. Subsequently, the sections were dehydrated, transparent, and sealed with neutral gum after drying slightly. Finally, slides were observed with microscopy (100X and 400X magnifications) for pathological evaluation.

### 2.10 Terminal deoxynucleotidyl transferase dUTP nick end labeling (TUNEL)

Paraffin‐embedded trophoblast sections were incubated at 60°C for 2 h, followed by dewaxing and rehydrating in xylene and graded ethanol (95%, 85%, and 75%) solutions. The sections were permeabilized by 0.1% Triton X‐100 (Beyotime) and then stained with TUNEL fluorescence staining reagents for 60 min at 37°C in the dark. After washing in PBS, the sections were coverslipped with 4′,6‐diamidino‐2‐phenylindole (DAPI) (Aladdin). For quantification, 3 different microscopic fields per section were counted and the percentage of TUNEL‐positive cells was determined.

### 2.11 Enzyme‐linked immunosorbent assay (ELISA)

The content of IL‐6 and TNF‐α was measured using the Mouse TNF‐α ELISA Kit, Mouse IL‐6 ELISA Kit, Human TNF‐α ELISA Kit, and Human IL‐6 ELISA Kit (LIANKE Biotech) according to the manufacturer's instructions. The protein concentration of the homogenate supernatant from mice tissues was determined by a BCA protein assay kit (Solarbio). All samples or standards were read on an ELISA plate reader at a wavelength of 450 nm and 570 nm. The difference between 450 nm and 570 nm absorbance values was the OD value after calibration. Finally, the corresponding concentration was calculated according to the standard curve.

### 2.12 Apoptosis detection by flow cytometry

Apoptosis assay was performed using an Annexin V‐FITC Apoptosis Detection Kit (KeyGen Biotech). Briefly, the transfected cells were washed twice with phosphate‐buffered saline (PBS) and re‐suspended in Binding Buffer. Then, cells were incubated with FITC‐conjugated Annexin V and PI for 15 min in the dark at room temperature. Finally, the samples were analyzed using flow cytometry. The apoptotic rate indicated the sum of late apoptotic cells (AnnexinV‐FITC+/PI+) and early apoptotic cells (AnnexinV‐FITC+/PI−).

### 2.13 Statistical analysis

GraphPad Prism software (version 8.0) was used to process the data and to perform statistical significance. Significance analysis was performed by unpaired *T*‐test and ordinary one‐way analysis of variance test. Experimental data were presented as the mean ± SD.

## 3 RESULTS

### 3.1 The expression of TMUB1 is upregulated in RSA patients

**Figure 1:**
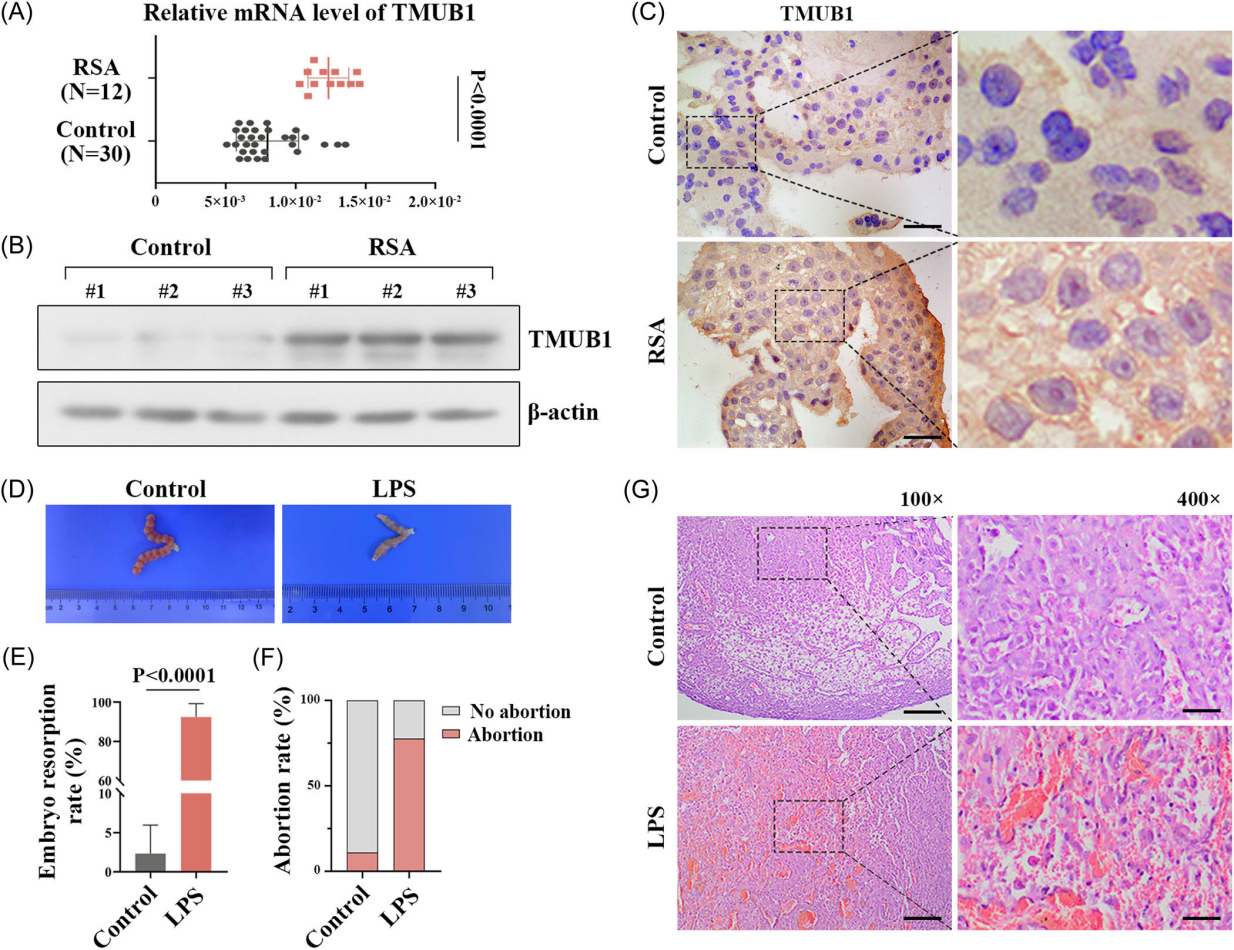
The expression of TMUB1 in recurrent spontaneous abortion (RSA) patients, and the pathological changes in LPS‐induced abortion mice (A and B) Quantitative RT‐PCR and western blot assays were used to detect the transcriptional and protein levels of TMUB1 in the villous tissues of pregnant women. (C) The expression of TMUB1 was detected in RSA women and patients with normal miscarriages by immunohistochemical analysis. Scale bars: 50 µm. (D) Pathological and anatomical observation of uteruses in pregnant mice. (E and F) The embryo absorption rate and abortion rate were calculated respectively in pregnant mice. (G) Histological analyses of hematoxylin‐eosin‐stained placenta tissues at magnification X100 (Scale bars: 200 µm) and magnification X400 (Scale bars: 50 µm). LPS, lipopolysaccharide; mRNA, messenger RNA; RT‐PCR, real‐time polymerase chain reaction; TMUB1, transmembrane and ubiquitin‐like domain containing 1.

To explore the effects of TMUB1 in RSA, we first detected the expression of TMUB1 in the placental villous tissues of aborted women. The results of Figure 1A indicated that the mRNA level of TMUB1 was significantly elevated in the placental villous tissues of RSA patients (*N* = 12) compared with the women who underwent induced abortions (*N* = 30). Western blot and IHC analysis also showed the upregulated expression of TMUB1 in the placental tissues of RSA patients (Figures 1B,C).

### 3.2 LPS induces pathological changes in placental villous tissues of pregnant mice

Next, we established the mouse model of abortion by LPS activation. Anatomical observations show that LPS‐treated mice have smaller uterine volumes and presented embryo loss than control mice (Figure 1D). The embryo resorption rate and abortion rate were markedly elevated after LPS stimulation (Figures 1E,F). H&E staining showed that placental villi appeared to be filled with blood and presented the nuclear disorder (Figure 1G).

### 3.3 LPS induces apoptosis and inflammation in placental tissues of pregnant mice

**Figure 2:**
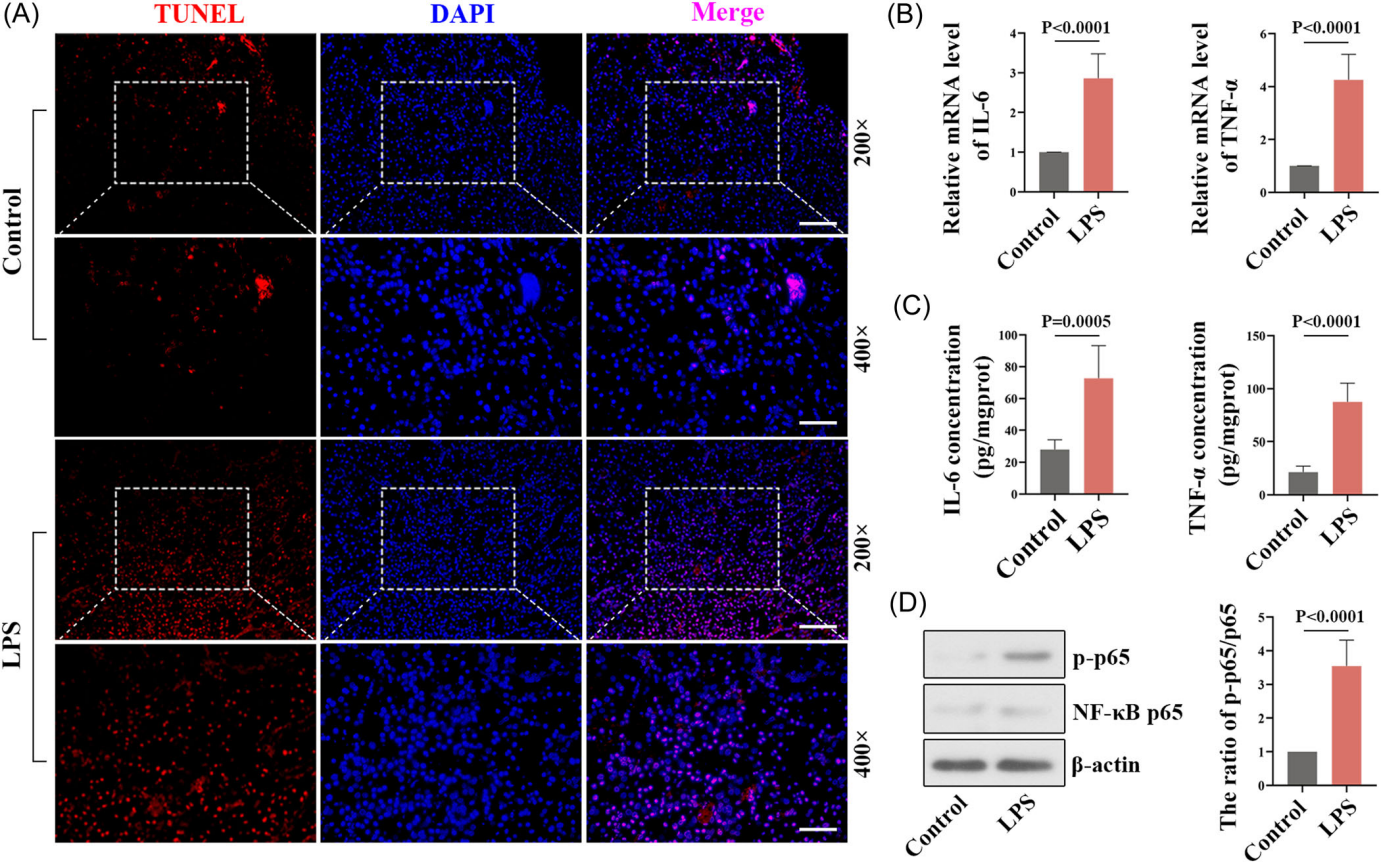
Effects of LPS on apoptosis and inflammation in placental tissues of pregnant mice (A) Representative images of TUNEL staining at magnification X200 (Scale bars: 100 µm) and magnification X400 (Scale bars: 50 µm). (B) The mRNA levels of IL‐6 and TNF‐α were measured with quantitative RT‐PCR. (C) The content of IL‐6 and TNF‐α was quantified by ELISA. (D) Western blot analysis for p‐p65 and p65 (left), and the quantification of the p‐p65/p65 ratio (right). ELISA, enzyme‐linked immunosorbent assay; IL‐6, interleukin‐6; LPS, lipopolysaccharide; mRNA, messenger RNA; RT‐PCR, real‐time polymerase chain reaction; TNF‐α, tumor necrosis factor‐α; TUNEL, terminal deoxynucleotidyl transferase dUTP nick end labeling.

TUNEL staining was used to investigate the effects of LPS‐treated pregnant mice on apoptosis. As shown in Figure 2A, the number of TUNEL‐labeled cells was increased in LPS‐activated mice compared with the control mice. The amounts of various cytokines in the placental tissues of mice were measured, and the results showed that the levels of IL‐6 and TNF‐α were significantly increased after LPS stimulation (Figures 2B,C). The protein amounts of p‐p65 (Ser536) and NF‐κB p65 in placental tissues were detected by western blot, and the results showed that LPS administration markedly lifted the ratio of p‐p65/p65 in pregnant mice (Figure 2D).

### 3.4 TMUB1 is highly expressed in the placental tissues of LPS‐induced mice

**Figure 3:**
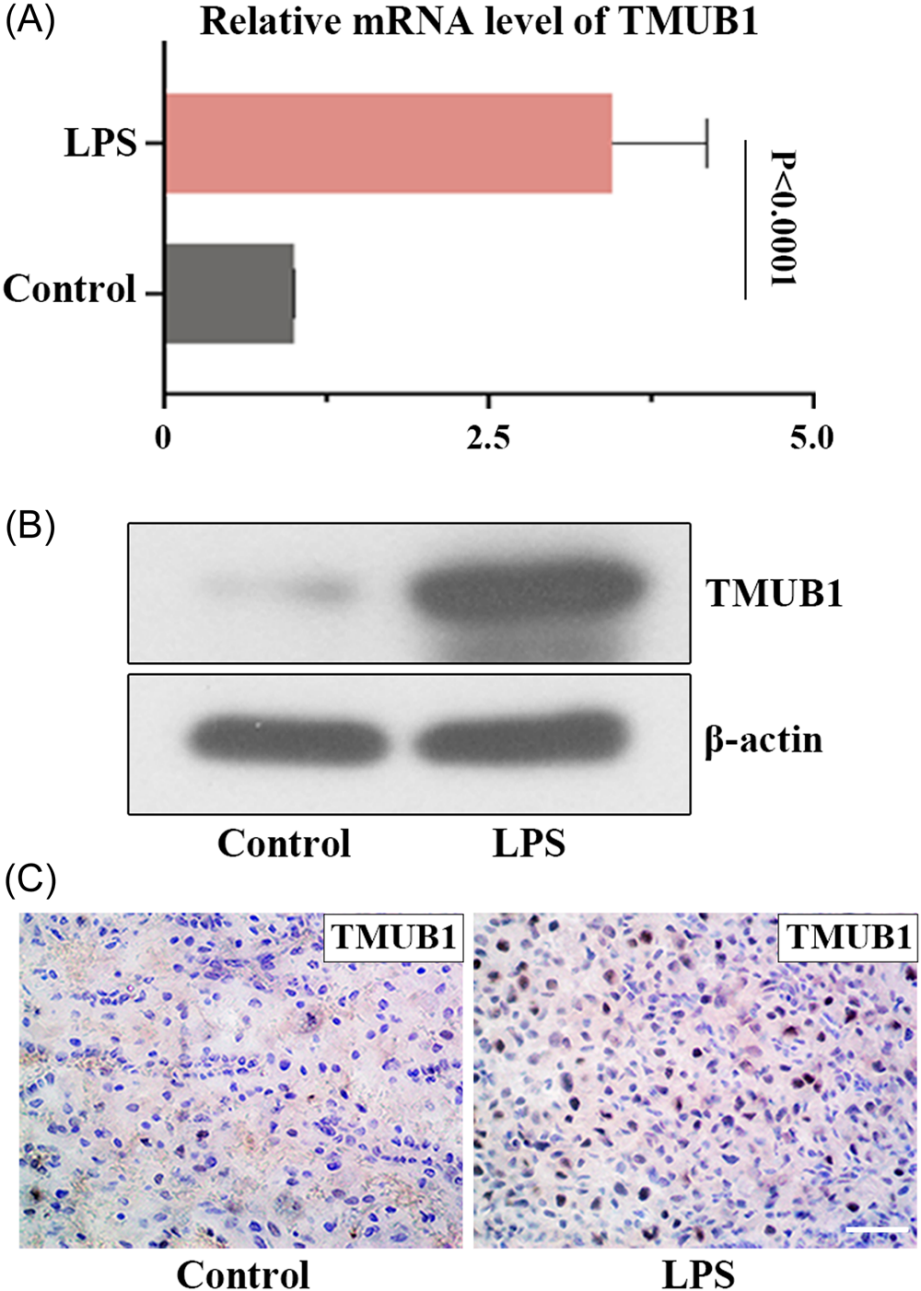
The expression of TMUB1 in placenta tissues of pregnant mice (A and B) Relative mRNA and protein levels of TMUB1 were detected by quantitative RT‐PCR and western blot. (C) The expression of TMUB1 in the placental villous tissues of LPS‐induced mice was detected by immunohistochemistry. Scale bars: 50 µm. LPS, lipopolysaccharide; mRNA, messenger RNA; RT‐PCR, real‐time polymerase chain reaction; TMUB1, transmembrane and ubiquitin‐like domain containing 1; TUNEL, terminal deoxynucleotidyl transferase dUTP nick end labeling.

To explore whether TMUB1 affected abortion, we assessed the expression of TMUB1 in LPS‐induced mice. We found that the mRNA and protein levels of TMUB1 were upregulated in the placental tissues of mice after LPS stimulation (Figures 3A,B). Immunohistochemical experiments showed that the pregnant mice treated with LPS also presented the upregulated expression of TMUB1 (Figure 3C).

### 3.5 TMUB1 knockdown attenuates apoptosis in LPS‐induced trophoblast cells

**Figure 4:**
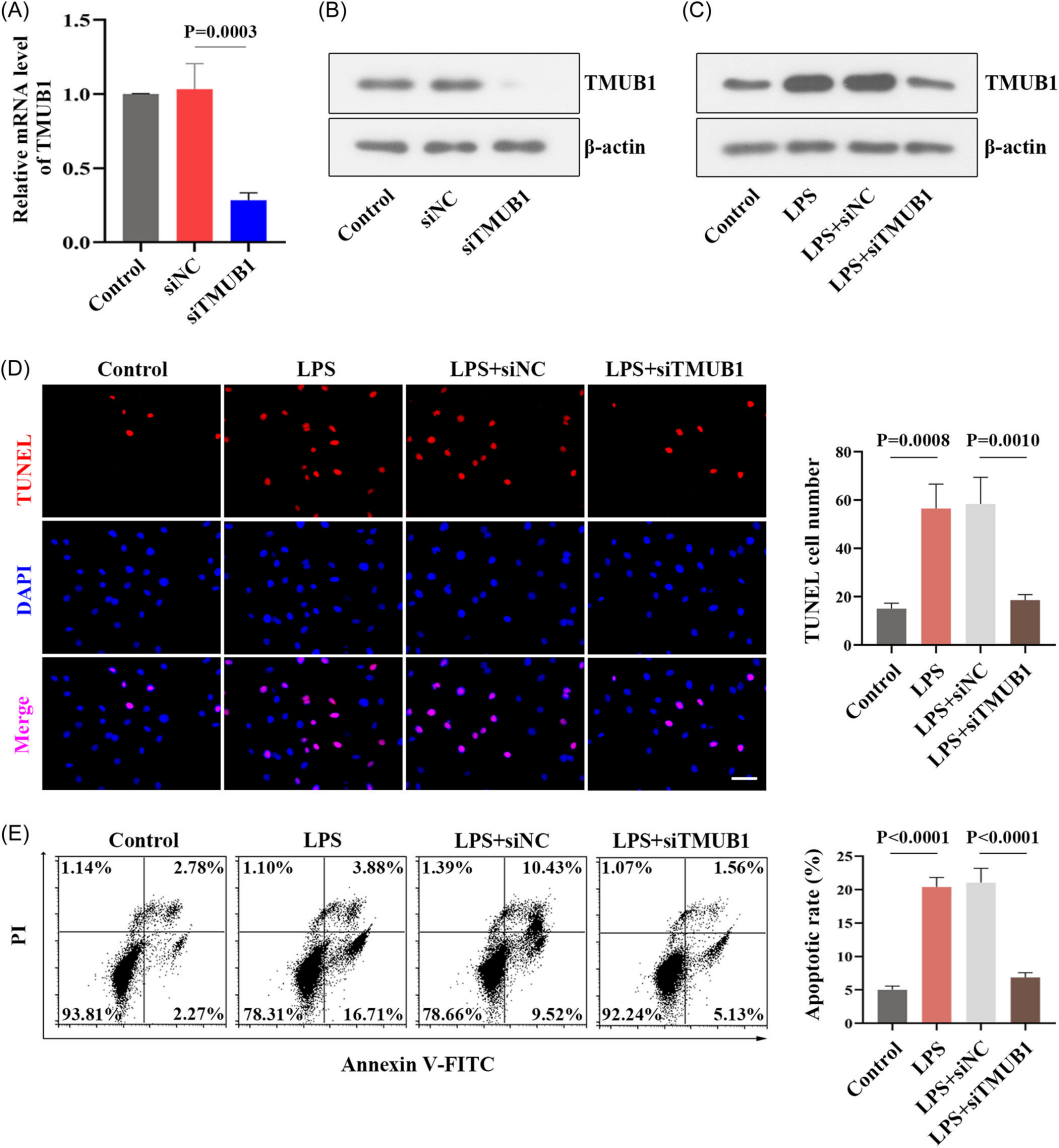
Effects of TMUB1 silencing on apoptosis in LPS‐induced trophoblast cells (A and B) The verification of TMUB1 knockdown efficiency was detected by quantitative RT‐PCR and western blot. (B) Western blot analysis for TMUB1 in TMUB1‐silenced cells after LPS activation. (D) TUNEL staining was used to detect apoptosis in LPS‐induced cells with TMUB1 knockdown. Scale bar: 50 µm. (E) The apoptosis rate (the sum of late apoptotic cells and early apoptotic cells) was analyzed using flow cytometry. AnnexinV‐FITC+/PI−: the early apoptotic cells; AnnexinV‐FITC+/PI +: the late apoptotic cells. annexin V‐FITC, annexin V‐fluorescein isothiocyanate; LPS, lipopolysaccharide; PI, propidium iodide; RT‐PCR, real‐time polymerase chain reaction; TMUB1, transmembrane and ubiquitin‐like domain containing 1.

To further analyze the function of TMUB1, we performed the loss‐of‐function experiments of TMUB1 in human trophoblast cells, and the transfected efficiency was verified by the mRNA and protein levels of TMUB1 (Figures 4A,B). After LPS activation, the protein amounts of TMUB1 were notably increased compared with the control group, and TMUB1 expression was downregulated in TMUB1‐silenced cells (Figure 4C). Compared with the LPS + siNC group, the number of TUNEL‐labeled cells was observably reduced in the LPS + siTMUB1 group (Figure 4D). Also, the apoptotic rate was lower in TMUB1‐silenced cells induced by LPS than in negative control cells (Figure 4E).

### 3.6 TMUB1 knockdown attenuates inflammation in LPS‐induced trophoblast cells

**Figure 5:**
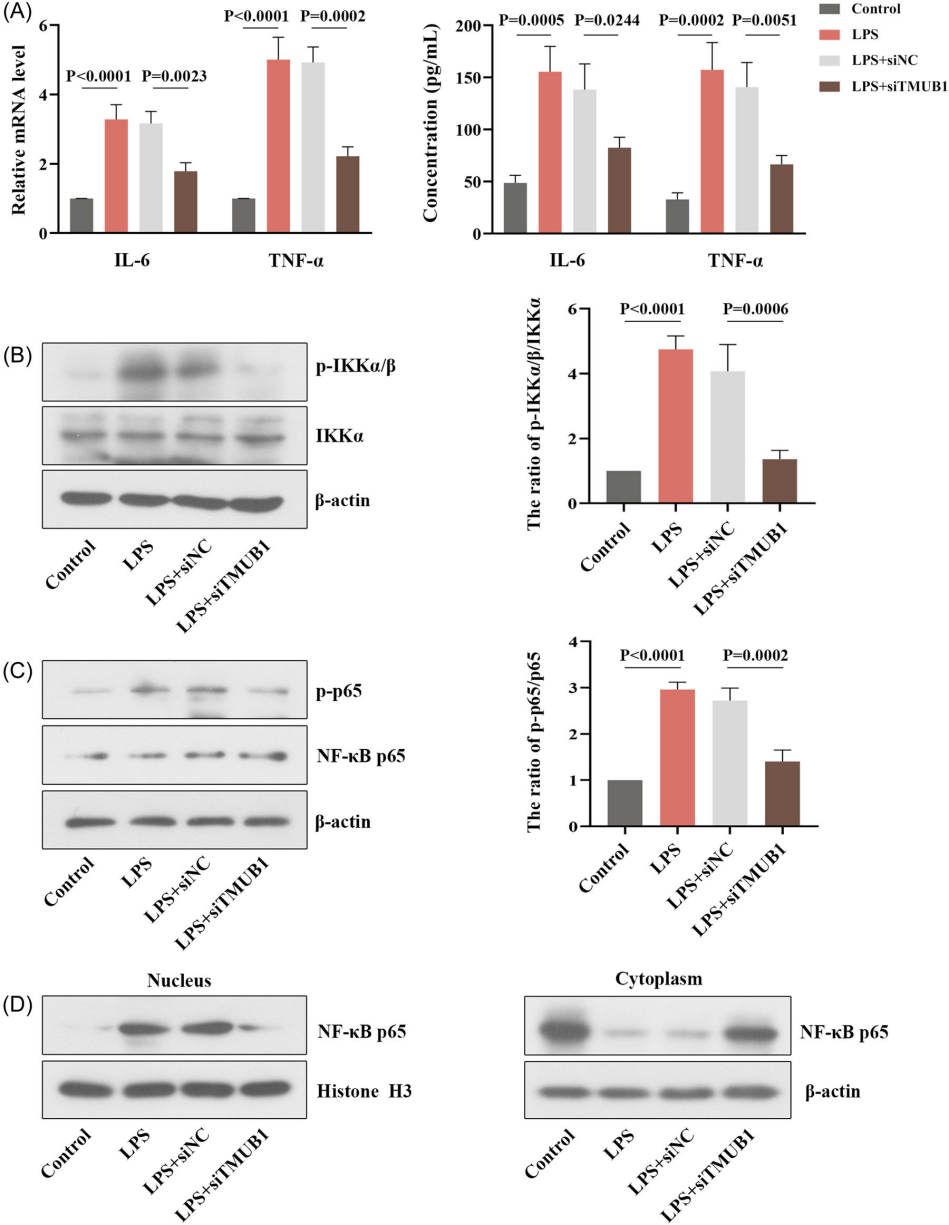
Effects of TMUB1 silencing on inflammation in LPS‐induced trophoblast cells. (B) The levels of IL‐6 and TNF‐α in the different treatments were measured by quantitative RT‐PCR and ELISA. (B) The expression of p‐IKKα/β and IKKα was detected by western blot, and the ratio of p‐IKKα/β/IKKα was quantified in different groups. (C) The expression of p‐p65 and p65 was detected by western blot, and the ratio of p‐p65/p65 was quantified in different groups. (D) The protein content of NF‐κB p65 in the cytoplasm and nucleus was detected by western blot. ELISA, enzyme‐linked immunosorbent assay; IL‐6, interleukin‐6; LPS, lipopolysaccharide; RT‐PCR, real‐time polymerase chain reaction; TMUB1, transmembrane and ubiquitin‐like domain containing 1; TNF‐α, tumor necrosis factor‐α.

The LPS‐induced trophoblast cells were used to explore the role of TMUB1 in inflammation. The levels of IL‐6 and TNF‐α were significantly dropped after TMUB1 knockdown (Figure 5A). After LPS activation, the loss of TMUB1 downregulated the protein expression of p‐IKKα/β, and the ratio of p‐IKKα/β/IKKα was decreased after TMUB1 silencing in LPS‐induced cells (Figure 5B). Western blot analysis indicated that the ratio of p‐p65/p65 was also decreased after TMUB1 knockdown (Figure 5C). As shown in Figure 5D, the protein expression of p65 in the nucleus was decreased in the LPS + siTMUB1 group, which elevated the protein content of NF‐κB p65 in the cytoplasm.

### 3.7 TMUB1 knockdown attenuates abortion in vivo

**Figure 6:**
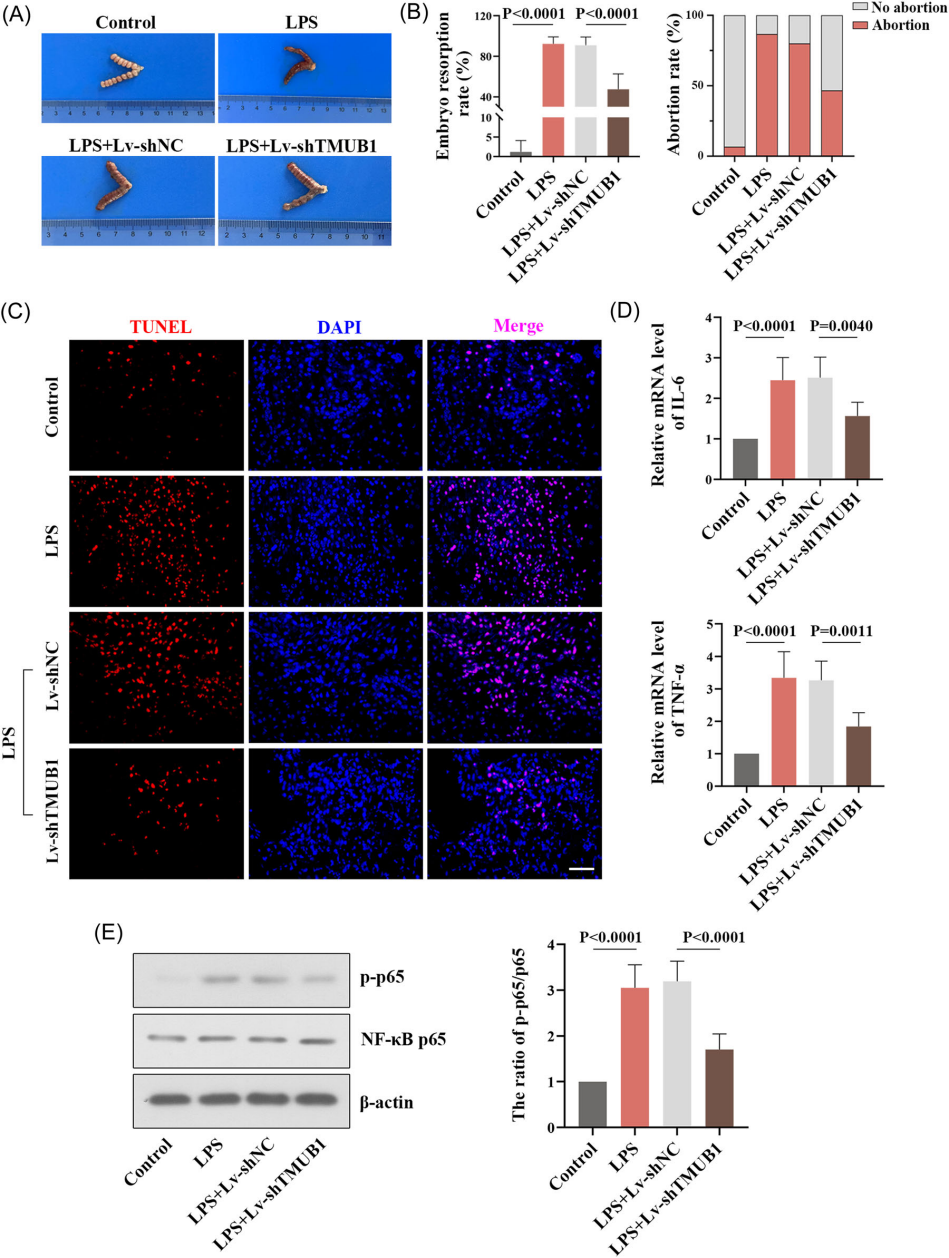
Effects of TMUB1 silencing in LPS‐induced mice. Pregnant mice were injected with lentivirus knocking down TMUB1 and then injected intraperitoneally with 0.15 μg/g LPS to induce abortion. (A) Pathological and anatomical observation of uteruses in pregnant mice. (B) The embryo absorption rate and abortion rate were calculated respectively in pregnant mice. (C) TUNEL staining was used to detect apoptosis in LPS‐induced mice with TMUB1 knockdown. Scale bar: 50 µm. (D) The mRNA levels of IL‐6 and TNF‐α were measured by quantitative RT‐PCR. (E) The protein content of p‐p65 and p65 was detected by western blot, and the ratio of p‐p65/p65 was quantified in different groups. DAPI, 4′,6‐diamidino‐2‐phenylindole; LPS, lipopolysaccharide; mRNA, messenger; RT‐PCR, real‐time polymerase chain reaction; TMUB1, transmembrane and ubiquitin‐like domain containing 1; TUNEL, terminal deoxynucleotidyl transferase dUTP nick end labeling.

Then the pregnant mice injected with lentivirus (Lv‐shTMUB1 or Lv‐shNC) were treated with LPS the following day. The observation of uteruses in Figure 6A showed that, compared with the control group, the embryos were lost and the uteruses exhibited high degrees of hyperemia in the LPS group, whereas lentivirus‐mediated knockdown of TMUB1 improved the deterioration of pathology caused by LPS. Moreover, we found that the loss of TMUB1 decreased the embryo resorption rate and abortion rate in LPS‐induced mice (Figure 6B). TUNEL staining showed that the number of apoptotic cells was reduced after TMUB1 knockdown (Figure 6C). In addition, we discovered that the mice silencing TMUB1 presented the decreased levels of IL‐6 and TNF‐α, as well as displayed the downregulated expression of p‐p65 after LPS activation (Figures 6D,E).

## 4 DISCUSSION

This study showed that TMUB1 was highly expressed in the villous tissues of RSA patients. The pathological alterations and the etiology of RSA induction (apoptosis and inflammation) were observed in LPS‐induced mice, and TMUB1 was found to be upregulated in mice after LPS activation. In vitro and in vivo experiments showed that TMUB1 downregulation significantly suppressed apoptosis and inflammation, along with inhibiting the NF‐κB expression and the cytoplasmic‐nuclear translocation of NF‐κB p65.

Cytokines are known to serve as the important determinants in pregnancy maintenance between the fetal and the maternal immune system.^7^ The accumulated evidence suggests that inflammatory cytokines are involved in LPS‐induced adverse pregnant outcomes,^19^, ^20^ and the levels of a series of inflammatory cytokines (such as TNF‐α, IFN‐γ, IL‐1β, IL‐27, and IL‐6) were markedly increased in the peripheral blood of the mice treated with LPS.^18^ The administration of LPS to pregnant mice causes placental degeneration due to acute inflammation, which results in fetal loss.^21^, ^22^ Here, we found that the embryo absorption rate and abortion rate were increased in LPS‐activated mice, which confirmed the validity of LPS as a potential inducer of inflammatory response and its associated impact on the placental vasculature.^23^, ^24^ Inflammation is reported to link with the pathogenesis of the female reproductive system which causes RSA.^25^ In this study, we found that TMUB1 silencing decreased the expression of inflammatory cytokines in LPS‐activated trophoblast cells, indicating the facilitating role of TMUB1 in the inflammatory response during the development of abortion. Additionally, apoptotic changes have been detected in the maternal‐fetal interfaces of the placenta during normal as well as complicated pregnancies.^26^ Another report also indicated that apoptotic changes in placenta tissue remodeling can cause the regular loss of placental trophoblast cells.^27^ Herein, the results showed that TMUB1 knockdown decreased apoptotic cells in LPS‐activated pregnant mice, which suggested the proapoptotic roles of TMUB1 in aborted mice. This result ties well with previous studies wherein TMUB1 promotes the p53‐dependent mitochondrial apoptosis pathway.^14^ Moreover, the researchers suggest that altered TMUB1 levels caused a significant defect in the induction of apoptosis, including reduced p53 protein levels and percentage of apoptotic cells.^28^

Phosphorylation of transcription factor is a fast and powerful mechanism that regulates transcription positively or negatively in many signal pathways, including Smads, c‐Jun, and NF‐κB.^29^, ^30^ In the case of NF‐κB, phosphorylation of Ser^536^ of the p65 subunit is the most common phosphorylation event associated with NF‐κB activation.^31^, ^32^, ^33^ Herein, we found that the loss of TMUB1 led to the ratio of p‐p65/p65 to fall in LPS‐activated cells, which suggested that TMUB1 might promote the inflammatory response via the NF‐κB pathway. Beyond that, NF‐κB, as a transcription factor, is reported to play a central role in many cellular events, including inflammation and apoptosis.^34^, ^35^ Emerging evidence indicates that the activation of NF‐κB primarily occurs via IKK‐mediated phosphorylation of inhibitory molecules.^36^ Here, we suggested that TMUB1 silencing significantly suppressed the phosphorylation of IKKα/β and p65, indicating that TMUB1 might play an important role in abortion through NF‐κB pathway‐mediated inflammation. Aban et al demonstrated that the upregulation of NF‐κB in the placenta was linked to apoptosis‐related markers, implying that trophoblast cell apoptosis is dependent on the NF‐κB pathway.^37^ The transcription factor NF‐κB may be implicated in the regulation of the response to embryopathic stressors, which cause excessive apoptosis.^38^ The crucial role of NF‐κB in the placenta and trophoblasts prompts us to investigate the biological function of TMUB1 in the activation of the NF‐κB pathway of RSA.

TMUB1 is actively exported from the hepatocyte nucleus in dividing cells, suggesting that TMUB1 is deemed as a nucleus‐cytoplasm shuttling protein.^12^ On this basis, our data from trophoblast cells with TMUB1 silencing suggested that TMUB1 decreased the phosphorylation of p65 and modulated NF‐κB activation. Consistent with our results, it has been reported that TMUB1 can enhance nuclear factor‐κB (NF‐κB) activation, thereby increasing the transcription of inflammatory mediators.^16^ Bellet et al. also mentioned the connection between TMUB1 and NF‐κB, and the experiments demonstrate that the transcriptional activity of the p65/RelA NF‐κB subunit is regulated by TMUB1.^16^ TMUB1 is demonstrated to inhibit the proliferation of rat liver cells and negatively modulate liver regeneration.^39^ Recent studies indicate that TMUB1 greatly facilitates antitumor immunity and inhibits tumor growth in mice, and it can serve as a potential candidate target to improve the prognosis of cancer‐immune patients.^40^ Our study demonstrated that TMUB1 knockdown blocked embryo loss and inhibited LPS‐induced inflammation and apoptosis. These findings indicate abnormal expression of TMUB1 is tightly linked to a series of illnesses and highlight the potential significance of TMUB1 as a biological marker. Beyond that, it is demonstrated that TMUB1 involves in the proliferation of hepatocellular carcinoma via the modulation of STAT1.^41^ Also, TMUB1 is reported to inhibit the phosphorylation of STAT3 and the activation of STAT3 signaling.^42^ These findings indicate that there is a putative feedback loop between TMUB1 and different signaling pathways.

In summary, our results confirmed that TMUB1 was highly expressed in the placental villous tissues of RSA patients. Further functional investigation suggested that TMUB1 knockdown attenuated inflammatory response and apoptosis in LPS‐induced trophoblast cells and mice. Moreover, the potential ability of TMUB1 in modulating NF‐κB activation was revealed by in vitro experiments. Taken together, we confirm that TMUB1 induces apoptosis and activates the NF‐κB pathway to respond to inflammation in the occurrence of abortion. Thus, TMUB1 may develop as a potential biomarker for RSA.

## AUTHOR CONTRIBUTIONS

**Xiuping Zhang**: Data curation; validation; writing—original draft. **Yuanjing Hu**: Conceptualization; data curation; validation. **Zhiping Zhang**: Validation. **Xueluo Zhang**: Validation. **Lixia Liang**: Validation. **Xiangrong Cui**: Data curation. **Yuanxia Wu**: Writing—original draft. **Fen Hu**: Writing—original draft. **Xueqing Wu**: Funding acquisition; Writing—review & editing.

## CONFLICT OF INTERESTS STATEMENT

The authors declare that there is no conflict of interest.

## ETHICS STATEMENT

This study was performed following the requirements of the Research Ethics Committee of Children's Hospital of Shanxi and Women's Health Center of Shanxi (IRB‐KYYN‐2021‐001).

## Data Availability

All data generated or analyzed during this study are included in this article.
